# Results of paclitaxel (day 1 and 8) and carboplatin given on every three weeks in advanced (stage III-IV) non-small cell lung cancer

**DOI:** 10.1186/1471-2407-5-10

**Published:** 2005-01-25

**Authors:** Perran F Yumuk, Nazim S Turhal, Mahmut Gumus, Nilgun F Hatabay, Orhan Turken, Alper Ozkan, Taflan Salepci, Mehmet Aliustaoglu, Rengin Ahiskali

**Affiliations:** 1 Division of Medical Oncology, Marmara University Hospital, Tophanelioglu C. 13/15 Altunizade, Uskudar, Istanbul 81190 Turkey; 2 Oncology Unit, Dr. Lutfi Kirdar Research and Training Hospital, Kartal, Istanbul, Turkey; 3 Oncology Unit, SSK Sureyyapasa Chest and Cardiovascular Diseases Hospital, Maltepe, Istanbul 34854 Turkey; 4 Division of Medical Oncology, Gulhane Military Medical Academy, Acibadem, Uskudar, Istanbul, Turkey; 5 Department of Pathology, Marmara University Hospital, Tophanelioglu C. 13/15 Altunizade, Uskudar, Istanbul 81190 Turkey

## Abstract

**Background:**

Both paclitaxel (P) and carboplatin (C) have significant activity in non-small cell lung cancer (NSCLC). The weekly administration of P is active, dose intense, and has a favorable toxicity profile. We retrospectively reviewed the data of 51 consecutive patients receiving C and day 1 and 8 P chemotherapy (CT) regimen in advanced stage NSCLC to evaluate the efficacy and toxicity.

**Methods:**

Patients treated in our institutions having pathologically proven NSCLC, no CNS metastases, adequate organ function and performance status (PS) ECOG 0–2 were given P 112.5 mg/m^2 ^intravenously (IV) over 1 hour on day 1 and 8, followed by C AUC 5 IV over 1 hour, repeated in every three weeks. PC was given for maximum of 6 cycles.

**Results:**

Median age was 58 (age range 39–77) and 41 patients (80%) were male. PS was 0/1/2 in 29/17/5 patients and stage was IIIA/IIIB/IV in 3/14/34 patients respectively. The median number of cycles administered was 3 (1–6). Seven patients (14%) did not complete the first 3 cycles either due to death, progression, grade 3 hypersensitivity reactions to P or lost to follow up. Best evaluable response was partial response (PR) in 45% and stable disease (SD) in 18%. Twelve patients (24%) received local RT. Thirteen patients (25%) received 2nd line CT at progression. At a median follow-up of 7 months (range, 1–20), 25 (49%) patients died and 35 patients (69%) progressed. Median overall survival (OS) was 11 ± 2 months (95% CI; 6 to 16), 1-year OS ratio was 44%. Median time to progression (TTP) was 6 ± 1 months (95% CI; 4 to 8), 1-year progression free survival (PFS) ratio was 20%. We observed following grade 3 toxicities: asthenia (10%), neuropathy (4%), anorexia (4%), anemia (4%), hypersensitivity to P (2%), nausea/vomiting (2%), diarrhea (2%) and neutropenia (2%). Two patients (4%) died of febrile neutropenia. Doses of CT were reduced or delayed in 12 patients (24%).

**Conclusions:**

P on day 1 and 8 and C every three weeks is practical and fairly well tolerated outpatient regimen. This regimen seems to be comparably active to regimens given once in every three weeks.

## Background

Lung cancer is the leading cause of cancer related deaths all around the world. About 80% of all lung cancers are non-small cell lung cancer (NSCLC) and more than 50% of these patients present with locally advanced or metastatic disease.

Meta-analysis of several randomized trials have demonstrated a modest survival advantage for treatment with cisplatin-based regimens in patients with advanced stages of NSCLC [1,2]. Furthermore, chemotherapy (CT) also has been shown to ameliorate symptoms and increase quality of life [3]. Addition of second generation CT regimens with cisplatin or carboplatin plus newer agents, such as taxanes (paclitaxel and docetaxel), gemcitabine, vinorelbine have shown increased response rates and 1-year survival ratios, but overall survivals have not been altered [4-6].

Being the first of the taxane antimicrotubule agents, paclitaxel (P) demonstrated overall response rates of 21–24% and 1-year survival rates of 37–42% in the phase II trials where it was used as a single agent [7,8]. Antiangiogenic effect of P has also been reported (9). Carboplatin (C) has also demonstrated comparable activity but better toxicity profile than cisplatin in the treatment of advanced NSCLC [10,11].

P and C used in combined chemotherapy regimens have significant activity in NSCLC. PC given every three weeks is considered to be one of the standard regimens being used worldwide [10]. The weekly administration of P is active, dose intense, and has a favorable toxicity profile. To evaluate the efficacy and toxicity of C and day 1 and 8 P in advanced stage NSCLC, we retrospectively reviewed 51 consecutive patients receiving this CT regimen.

## Methods

All patients with stage III or IV NSCLC treated at Medical Oncology Units of Marmara University Hospital, Dr. Lutfi Kirdar Research and Training Hospital, SSK Sureyyapasa Chest and Cardiovascular Diseases Hospital and Gulhane Military Medical Academy Hospital within July 2002 and August 2003 were considered for this protocol. Eligible patients were required to have pathologically proven NSCLC, stage III or IV disease at presentation or progressed after surgery, performance status (PS) ECOG 0–2, objective measurable disease, adequate bone marrow functions (white blood cell count ≥ 3500/mm^3^, hemoglobin ≥ 9 g/dl, and platelet count ≥ 100000/mm^3^), and adequate liver functions (bilirubin ≤ 1.5 mg/dl and alanine aminotransferase ≤ 2 times upper limit of normal and ≤ 5 times upper limit of normal for the patients with liver metastases) and kidney functions (creatinine ≤ 1.5 mg/dl). No prior chemotherapy or radiotherapy (except to bone metastases for palliation) was allowed. Patients presenting with known central nervous system (CNS) disease and uncontrolled cardiac arrhythmia were excluded from this study and they were treated with other chemotherapy regimens (single agent or combination of platinum and vinorelbine, docetaxel or gemcitabine).

Patients were treated with P (112.5 mg/m^2^/day) on days 1 and 8, followed by C (AUC 5/6) on day 1, repeated in every three weeks. Both drugs were diluted in 250 ml of normal saline and given intravenously (IV) over 1 hour. No growth factors were administered. Anti-allergic premedication included IV diphenhydramine 50 mg, IV ranitidine 50 mg, and IV dexamethasone 16 mg 1 hour prior to P administration.

Toxicity evaluation and routine physical examination were performed in every 3 weeks. Complete blood count (CBC) was done on days 1 and 8 of each cycle, liver and kidney function tests on every 2 cycles. Cranial computed tomography scans (CT), magnetic resonance imaging (MRI) and bone scans were performed as clinically indicated. Side effects of the treatment were graded according to the National Cancer Institute Common Toxicity Criteria (CTC), version 2.0 [12]. Colony stimulating factors were not used.

Response was evaluated with CT of chest and/or abdomen on every 3^rd ^cycle and standard World Health Organization (WHO) criteria were used to determine response [13]. Independent of the stage at presentation, patients having partial response (PR), stable disease (SD) or progressive disease (PD) during CT were consulted for radiotherapy (RT) for either primary treatment or palliation. The treatment was stopped for patients with PD. Patients with CR, PR or SD after 3 cycles continued their treatment. PC was given for maximum of 6 courses to the patients having PR or SD.

Patients were irradiated with CT based treatment planning and multiple fields arrangements with custom blocking to all fields and involved hilar and mediastinal lymph nodes up to 40–41.4 Gy. Boost was given to the primary tumor. Total dose of 60–61.2 Gy was administered in 1.8–2 Gy daily fractions for 5 days a week and completed in 6 weeks.

### Statistical analysis

Overall survival (OS) and time to progression (TTP) were assessed from the date of diagnosis to the date of death (any cause) and the date of objective disease progression (death was considered a progression event in patients who died before disease progression), respectively. Survival rates were calculated by using the Kaplan-Meier method [14]. The pre-specified prognostic value of age (< 60 years vs. ≥ 60 years), gender, PS (0 vs. 1–2), histology (adenocarcinoma vs. squamous cell vs. NSCLC), stage (III vs. IV), smoking history (present vs. absent), and response after third cycle of CT (PR vs. other) were evaluated in univariate and multivariate analyses. Log rank test was used for univariate survival analysis [15]. The multivariate Cox proportional hazard model was applied to identify the variables that can independently influence survival.

## Results

The data of 51 patients receiving PC treatment were collected retrospectively between July 2002 and November 2003. Median follow-up time was 7 months (range, 1–20). Median age was 58 years (range 39–77) and 45% of patients were 60 year-old or above. Eighty percent were male. PS was 0 in 57% of patients and 67% had presented with stage IV disease. Most frequent metastatic sites were the other lung (17), adrenal (10), liver (7) and bone (7). Eighty-two percent of the patients had smoking history, median of which was 40 pack-years (range, 0–135). Patients' baseline characteristics are presented in Table 1.

The median number of cycles administered was 3 (range, 1–6). Seven patients (14%) did not complete the first 3 cycles either due to death (2), progression (3), grade 3 hypersensitivity reaction to P (1) or lost to follow up (1).

Best evaluable response was PR in 45% and SD in 18%. Only 22 (43%) patients continued the treatment after the 3rd course. At the end of treatment of these 22 patients 10 (46%) had PR and 6 (27%) had SD, but the other 6 patients (27%) had PD. No complete remission was seen. Twelve patients (24%) received local RT and 4 of these patients were given low dose gemcitabine (75 mg/m2/week × 5–6 weeks) as radiosensitizing agent. Of these 12 patients 3 presented with stage IIIA and all had PR to PC therapy. But of the 5 patients with IIIB disease who were irradiated only one patient had PR, 3 had SD and another one had PD after the 3 cycles of CT. Four patients with stage IV were offered RT for palliation. Thirteen patients (25%) received 2nd line CT at progression and of those only one patient had PR and another SD to this treatment. For 2nd line CT gemcitabine ± cisplatin or C was used in 10 patients (78%) and the rest received other agents like vinorelbine, C or docetaxel. Details of this data can be seen in Table 2.

**Figure 1:**
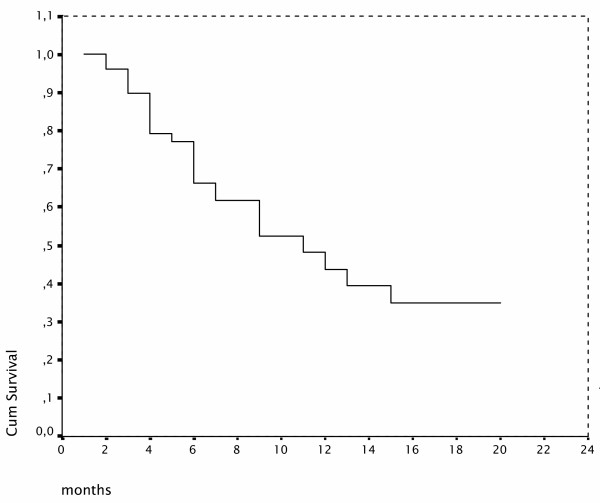
Overall survival Kaplan-Meier curve

**Figure 2:**
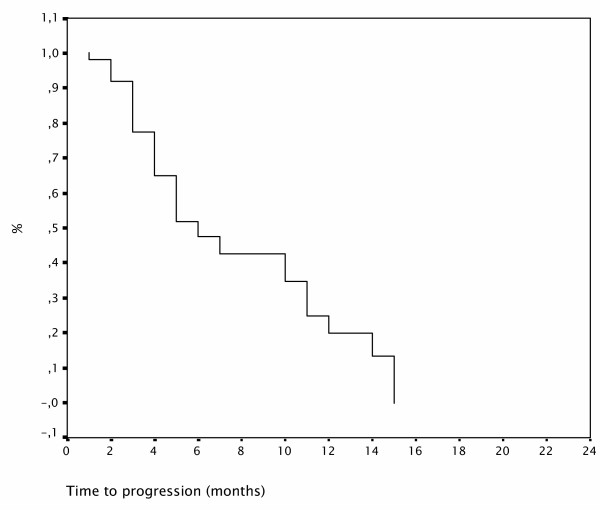
Progression free survival curve

At a median follow-up of 7 months 25 (49%) patients died and 35 patients (69%) progressed. Median OS time was 11 ± 2 months (95% CI; 6 to16), 1-year OS ratio was 44% (Figure 1). Median TTP was 6 ± 1 months (95% CI; 4 to 8), 1-year progression free survival (PFS) ratio was 20% (Figure 2).

The most frequent toxicity related symptoms were asthenia (61%), neuropathy (42%) and anorexia (35%). We observed the following grade 3 toxicities: asthenia (10%), neuropathy (4%), anorexia (4%), anemia (4%), hypersensitivity to P (2%), nausea/vomiting (2%), diarrhea (2%) and neutropenia (2%). Two patients (4%) died of febrile neutropenia due to a three day delay in referral to hospital after the onset of fever > 38°, although they were warned about the side effects of the therapy. Doses of CT was reduced or delayed in 12 patients (24%) (Table 3).

Univariate analysis showed that patients presenting with PS of 0, stage III disease and having PR after the 3rd cycle of PC have statistically higher OS (p = 0.015, p = 0.018 and p = 0.047, respectively)(Table 4). PS and stage of the disease at presentation and response to the CT after the 3rd cycle were also statistically significant independent prognostic factors influencing the OS in multivariate Cox regression analysis (p = 0.034, p = 0.049 and 0.021, respectively).

## Discussion

Paclitaxel and carboplatin have been shown to be an effective and well tolerated CT regimen in advanced stage NSCLC [10]. PC given once in every three weeks is one of the most widely used standard schedules worldwide based on the spectrum of activity and the ease of administration. This regimen results in an objective response rate of 17–25% with a median survival time of 8 months in stage IIIB and IV NSCLC patients. The major toxicities of this regimen are neuropathy and neutropenia [10,16].

Weekly P is a relatively new strategy for lowering toxicity and increasing dose-intensity and possibly efficacy. Alvarez et al. have used weekly P on patients who progressed or remained stable on P administered in every three weeks and reported that it can induce response in 62.5% of patients with low toxicity [17]. Akerley has also studied weekly P administration on phase I and phase II settings [18-20]. They started with a P dose of 175 mg/m^2^/week × 6 every 8 weeks in the phase II trial, but they had to reduce the dose up to 50% due to primarily neutropenia and neuropathy with extended therapy. Therefore, they recommended 150 mg/m^2 ^as the weekly dose of P [20].

Weekly dose of P in combination with cisplatin or C had been administered in NSCLC patients by Belani et al. [21,22]. They used this combination in a multicenter three arm trial in 401 patients with stage IIIB and IV disease [21]. In that trial P was given 100 mg/m^2^/week for 3 weeks out of 4 week cycles in arms I and II, with C either AUC of 6 on day 1 or AUC of 2 on days 1, 8 and 15 of each of four 4-week cycles. Arm III of this trial consisted of P (150 mg/m^2^) and C (AUC = 2) given weekly for 6 out of 8 weeks for a total of two cycles. Greater percentage of the patients on arm I received intended CT (30% of P and 55% of C) compared with the other arms (28–29% of P and 21–22% of C). Patients on arm I received more than half of the planned C dose. The main reasons for discontinuation of therapy were progression of disease (31%) and adverse events (15%). Median time to progression and median survival time were significantly higher for arm I than arm II for patients with stage IIIB disease. Performance status of the patients was also statistically related to the survival times. Patients with PS-0/1 had longer median PFS with treatment arm I than arm II and patients with PS-2 had higher median OS with arm I than arm II. Although arm I was the most easily tolerable schedule between the three arms, grade 3 or 4 neutropenia was observed in 22% of the patients included. In this trial treatment arm I had a response rate of 32%, median TTP of 6.9 months, median OS time of 11.3 months and 1-year survival rate of 47%. In our study, response rate was 45%, median TTP was 6 months, median OS time was 11 months and 1-year survival rate was 44%. The majority of our patients comprised of stage IIIB and IV disease, similar to the patient group in Belani's study resulting in similar response rates and survival data [21]. These results also seem more effective than the regimen given once in every three weeks of the same drugs [10,16].

We used the standard dose of P (225 mg/m^2^) given in every three weeks and divided into two consecutive weeks. C dose was calculated according to Calvert formulation with an AUC of 5. This is a lower dose than the dose of C being used in other phase III trials in the literature. In our study only 4 patients (8%) had dose reduction of 10% and 16% of patients had treatment delays of 1 week because of side effects. According to this data, 76% of patients have received the total planned doses of the drugs on scheduled date. Two patients (4%) died of febrile neutropenia due to a three day delay in referral to hospital after the onset of fever > 38°, although they were warned about the side effects of the therapy. It is worth mentioning that none of our patients received any colony stimulating factors.

We have shown that the response to treatment after the third cycles of CT was one of the independent prognostic factors influencing OS. It has already been reported by Socinski et al. that 4 cycles of CT give the maximum benefit which could be obtained from CT in patients with stage IIIB and IV NSCLC [23]. Smith et al. also studied 3 cycles versus 6 cycles of CT in the same group of patients and failed to show any survival advantage for longer treatment durations [24]. In addition, there was an increase in the side effects such as fatigue, nausea and vomiting in the patients receiving six courses.

It has been shown that PC combination has relatively mild toxicity profile. Belani et al. observed in their phase I trial that patients who received the PC combination in every three weeks experienced less severe thrombocytopenia than would be expected from C alone. In the view of this finding they suggested that there was a platelet-sparing effect of P on the dose-limiting thrombocytopenia side effect of C [25]. This phenomenon was also shown by Akerley [18] and Kearns [26]. Akerley reported that platelet counts rose by 17000/mL/week with weekly P administration [18]. Belani also speculated on the mechanism for this platelet protective effect and said that it may involve some alteration of megakaryocytopoiesis or thrombocytopoiesis, which could result in increased levels of endogenous thrombopoietin or other cytokines [27]. Kearns et al. suggested that prior exposure to P may suppress the inhibition of platelet formation, which is associated with C [26]. None of our patients experienced thrombocytopenia during our CT treatment with day 1 and 8 P with day 1 C on every three weeks.

One of the most frequent side effects during our treatment was neuropathy, but it was usually mild (Grade 1 or 2), only 4% of our patients experiencing grade 3 sensory neuropathy. Grade 3 or 4 neuropathy has been reported to be 10–20% in schedules given every three weeks [10,16]. Belani reported 3–13% of grade 3 or 4 neuropathy, but the incidence was lower for arms 1 (P given weekly and C every four weeks) and 2 (P and C both given weekly), at only 5% and 3%, respectively [21]. This result for arm 1 is similar to the neuropathy rate in our study.

Besides the reduced toxicity, weekly administration of P also increases the drugs' anti-angiogenic and apoptotic effects. The metronomic schedule of P has been studied widely during the last few years. P had been shown to inhibit endothelial cell proliferation, motility, invasiveness, and cord formation both in vitro and in vivo Matrigel assays in a dose dependent manner [9]. Belani et al. randomized the patients having objective response to weekly P and C regimens into two arms (maintenance and observation arms). Patients were either treated with weekly P (70 mg/m^2^/week × 3 weeks out of four weeks cycles) in maintenance arm or observed until disease progression has occurred. They reported that the maintenance arm was compared to the observation arm and had a median PFS of 38 weeks vs. 29 weeks, median OS of 75 weeks vs. 60 weeks, respectively [21]. Although there was not a statistically significant difference between the two arms, authors concluded that this might be a result of low number of patients enrolled in the study (only 65 patients in each arm). It is not yet known whether these responses have an anti-angiogenic basis, or whether such responses will translate into a significant prolongation of survival.

Although our study is a retrospective analysis, it is one of the few manuscripts on this PC scheduling in NSCLC in the literature.

## Conclusions

Paclitaxel on day 1 and 8 and carboplatin every three weeks is a practical and fairly well tolerated outpatient regimen. This regimen seems to be comparably active to regimens given every three weeks. This schedule needs to be further evaluated by well planned randomized phase III trials where it could be compared to the standard regimens in patients with advanced stage NSCLC.

## Competing interests

The author(s) declare that they have no competing interests.

## Authors' contributions

PFY designed the study, followed the patients, collected the data, performed the statistical analysis and drafted the manuscript. NST followed the patients and helped with the manuscript. MG followed the patients and helped with statistical analysis. NFH, OT, AO, TS, MA followed the patients. RA confirmed the diagnosis. All authors read and approved the final manuscript.

## Pre-publication history

The pre-publication history for this paper can be accessed here:



## Figures and Tables

**Table 1:** Patient characteristics

Characteristics	Number of patients	Percentage (%)
Age, years		
Median	58	
Range	(39–77)	

Gender		
Male	41	80
Female	10	20

Performance Status		
0	29	57
1	17	33
2	5	10

Stage		
IIIA	3	6
IIIB	14	27
IV	34	67

Histology		
Adenocarcinoma	16	31
Squamous cell carcinoma	18	36
NSCLC	17	33

Sites of metastases		
Lung	17	50
Adrenal	10	29
Bone	7	21
Liver	7	21
Distant LAP	3	9

Number of metastatic organs		
1	24	71
2	9	26
3	1	3

Smoking history		
Yes	42	82
No	9	18

**Table 2:** Response rates and second line treatments with CT and RT

	Number of patients	Percentage (%)
Number of PC received		
1	2	4
2	5	10
3	20	39
4	3	6
5	3	6
6	18	35

Response to PC		
PR	23	45
SD	9	18
PD	16	31
Not assessed	3	6

Second line CT	13	
G ± Cis / C	10	78
Other	3	23

Local RT	12	
Stage IIIA	3	25
Stage IIIB	5	42
Stage IV	4	33

CT: Chemotherapy; RT: Radiotherapy; P: Paclitaxel; C: Carboplatin; G: Gemcitabine; Cis: Cisplatin

**Table 3:** Toxicities seen during PC treatment

Toxicity	Overall (%)	Grade 1–2 (%)	Grade 3 (%)	Grade 4 (%)
Neutropenia	12	10	2	
Febrile neutropenia	4			4
Anemia	22	18	4	
Asthenia/Fatigue	61	51	10	
Neuropathy	42	38	4	
Anorexia	35	31	4	
Arthralgia	31	31		
Nausea/Vomiting	22	20	2	
Diarrhea	8	6	2	
Mucositis	6	6		
Hypersensitivity to P	2		2	

**Table 4:** Prognostic factors in the univariate analyses for overall survival

**Variables**	Median OS (months)	p
Age		
60 years and over	11 ± 2	0.87
Less than 60 years	12 ± 3	
Gender		
Male	9 ± 3	0.22
Female	15 ± 6	
PS		
0	15 ± 3	0.015*
1–2	6 ± 2	
Histology		
Adenocarcinoma	15 ± 5	0.73
Squamous cell	13 ± 7	
NSCLC	11 ± 3	
Stage at presentation		
III	NR^†^	0.018*
IV	9 ± 2	
Smoking history		
Yes	9 ± 2	0.20
No	NR^†^	
Response after 3 cycles of CT		
PR	13 ± 2	0.047*
Other	6 ± 1	

* Statistically significant

† Mean OS is 15 ± 2 months

PS: ECOG Performance status; NR: Not reached; CT: Chemotherapy
